# A2 USING BIOPSY-DERIVED ORGANOIDS AND GUT-ON-A-CHIP PLATFORM TO DEVELOP A PHYSIOLOGICALLY RELEVANT BACTERIAL INFECTION MODEL

**DOI:** 10.1093/jcag/gwae059.002

**Published:** 2025-02-10

**Authors:** Q Liang, K Choi, H Sham, B Bressler, B vallance

**Affiliations:** Experimental Medicine, University of British Columbia, Vancouver, BC, Canada; Experimental Medicine, University of British Columbia, Vancouver, BC, Canada; BC Children’s Hospital Research Institute, Vancouver, BC, Canada; Experimental Medicine, University of British Columbia, Vancouver, BC, Canada; Experimental Medicine, University of British Columbia, Vancouver, BC, Canada

## Abstract

**Background:**

The intestine is a highly complex ecosystem, consisting of diverse cell types, dynamic interactions, and mechanical forces. These elements are key to maintaining intestinal homeostasis and modulating the risk of enteric bacterial infections. Traditional *in vitro* models fail to recapitulate the physiological complexity of the gut, limiting our understanding of bacterial pathogenesis and host responses. Patient-derived intestinal organoids offer a promising solution by replicating the cellular diversity and structure of the epithelium. However, conventional organoid cultures lack the dynamic environment present *in vivo*. The emergence of organ-on-a-chip technology provides an innovative approach for such modeling, by incorporating mechanical forces, such as flow and stretch, to better mimic the intestinal environment.

**Aims:**

To establish a bacterial infection model using patient-derived intestinal organoids cultured in a gut-on-a-chip system to address the challenges of simulating the complex enteric pathogen-host interactions.

**Methods:**

An enteric *Salmonella* infection model was developed using the Emulate Colon Intestine-Chip. This system consists of two parallel channels seeded with patient-derived ascending colonic organoids in the upper channel and Human Intestinal Microvascular Endothelial Cells (HIMEC) in the lower. Cellular differentiation, barrier integrity, mucus and cytokine release, and pathogen invasion was assessed.

**Results:**

Biopsy-derived colonic organoids rapidly differentiated into a multi-cell type system when co-cultured with HIMEC on the Emulate Chip, showing tight junction formation and a stable epithelial barrier. Following *Salmonella* infection, a significant decrease in intestinal permeability was observed in infected organoids compared to uninfected controls, indicating a breach in epithelial integrity. This disruption was accompanied by release of inflammatory cytokines, including CCL2 and IL-8, demonstrating a robust innate immune response. Notably, goblet cell release of mucus into the supernatant were observed post-infection, suggesting a rapid secretion of mucus as a defense mechanism against infection. Microscopy confirmed extensive *Salmonella* invasion into the epithelial layer, demonstrating the organ-on-a-chip system’s ability to replicate key aspects of enteric infection as observed seen *in vivo*.

**Conclusions:**

The *Salmonella* infection model established using the gut-on-a-chip platform effectively recapitulates key features of enteric infection within a physiologically relevant microenvironment. This model demonstrates the loss of epithelial integrity, the activation of robust inflammatory responses, and rapid mucus secretion, offering a powerful tool for studying host-pathogen interactions in the human gut and potentially informing therapeutic development for enteric diseases.

**Funding Agencies:**

Weston Family Foundation

